# Comparing the Psychological Effects of Meditation- and Breathing-Focused Yoga Practice in Undergraduate Students

**DOI:** 10.3389/fpsyg.2020.560152

**Published:** 2020-11-12

**Authors:** Xin Qi, Jiajin Tong, Senlin Chen, Zhonghui He, Xiangyi Zhu

**Affiliations:** ^1^Department of Physical Education and Research, Peking University, Beijing, China; ^2^Beijing Key Laboratory of Behavior and Mental Health, School of Psychological and Cognitive Sciences, Peking University, Beijing, China; ^3^School of Kinesiology, Louisiana State University, Baton Rouge, LA, United states

**Keywords:** meditation, breathing, yoga, work intention, mindfulness, stress

## Abstract

**Objectives:**

The present study aimed to compare the psychological effects of meditation- and breathing-focused yoga practice in undergraduate students.

**Methods:**

A 12-weeks yoga intervention was conducted among a group of undergraduate students enrolled in four yoga classes at an academically prestigious university in Beijing, China. Four classes were randomized to meditation-focused yoga or breathing-focused yoga. A total of 86 participants finished surveys before and after the 12-weeks intervention, measuring work intention, mindfulness, and perceived stress. The repeated-measure multivariate analysis of covariance (MANCOVA) followed by univariate analyses were conducted to examine the differences in work intention, mindfulness, and stress between the two yoga intervention groups over the semester, after controlling for age and gender.

**Results:**

The repeated-measure MANCOVA revealed significant group differences with a median effect size [Wilks’ lambda, Λ = 0.90, *F*(3, 80) = 3.10, *p* = 0.031, η^2^ = 0.104]. Subsequent univariate analyses showed that students in the breathing-focused yoga group had significant higher work intentions [*F*_(1, 82)_ = 5.22; *p* = 0.025; η^2^_*p*_ = 0.060] and mindfulness [*F*_(1, 82)_ = 6.33; *p* = 0.014; η^2^_*p*_ = 0.072] but marginally lower stress [*F*_(1, 82)_ = 4.20; *p* = 0.044; η^2^_*p*_ = 0.049] than students in the meditation-focused yoga group.

**Conclusion:**

Yoga practice with a focus on breathing is more effective than that with a focus on meditation for undergraduates to retain energy for work, keep attention and awareness, and reduce stress.

## Introduction

Yoga has different components including postures, movements, meditation, and breathing (pranayama) (Brown and Gerbarg, 2005). The current literature has seen a general consensus of yoga’s function in retaining energy and vitality (Bowden et al., 2012; Tyagi et al., 2016) and reducing stress (Riley and Park, 2015). However, little is known of the relative importance of specific components of yoga practices, especially between the most revisited practices of meditation and breathing. Prior research has shown that yoga postures and movements can reduce depression (Carter and Byrne, 2004), but meditation and breathing may be more important to other mental health outcomes including stress and mood (Wheeler et al., 2019), sustained attention (Schmalzl et al., 2018), working memory capacity (Quach et al., 2016) posttraumatic stress disorder, hyperarousal symptoms of sleep disturbance, flashbacks, or anger outbursts (Carter and Byrne, 2004). Moreover, there are evidences for potential differences between meditation and breathing in literature such as the levels of energy (Joshi and Telles, 2009; Zaccaro et al., 2018), attention, and practice difficulty (Brown and Gerbarg, 2009). Thus, the yoga practices of meditation and breathing may function differently in a group of novices with daily mental activities. Our findings may help disclose the nature of yoga components and provide unique guidance for novices with mental work in yoga practices.

Meditation refers to engagement of mental exercise to reach a heightened level of spiritual awareness (Clarke et al., 2018). Meditation is frequently reported to have significant impacts on biological and psychological outcomes. For example, Hendriks (2018) systematically reviewed 11 research studies to examine the effects of yoga meditation on mental health outcomes such as anxiety, depression, stress, and well-being. Meditation also has been studied as an independent practice beyond the scope of yoga such as Buddhist meditation, compassion meditation, mindfulness meditation, and sound meditation. For example, one study showed that meditation was associated with molecular changes in cerebral cortex, prefrontal area, autonomic nervous system, hormones, etc. (Jindal et al., 2013). Meditation is beneficial to cognitive outcomes (Chiesa et al., 2011) and can facilitate stress management (Borchardt and Zoccola, 2018). Specifically, traditional Buddhist meditation programs led to improvement in stress levels and mood (Shonin et al., 2014). Gallegos et al.’s (2017) and Hilton et al.’s (2017) meta-analysis found that mindfulness meditation alleviated the symptoms of posttraumatic stress. Wielgosz et al.’s (2019) review summarized the efficacious applications of mindfulness meditation to many specific domains of psychopathology. Philips et al. (2019) found that sound meditation was better than silence meditation to relax and reduce acute stress.

Breathing as a mental practice has received increasing research interest. During yoga exercise, an individual can practice different patterns of breathing and use specialized techniques to enhance breathing skills such as inhaling deeply into the abdomen, holding the breath at certain parts of the breathing cycle (Brown and Gerbarg, 2009), breathing at varying rates, such as *Sudarshan Kriya* yogic breathing (Brown and Gerbarg, 2005), and/or high-frequency yoga breathing (Joshi and Telles, 2009). Brandani et al.’s (2017) review of 13 studies showed that yoga breathing had a hypotensive effect. Joshi and Telles (2009) found that high-frequency yoga breathing improved selective attention and that breath awareness increased available neural resources by examining event-related potentials (i.e., the P300). Besides biological impacts, breathing, as an important component of yoga practice, can bring upon dramatic psychological and mental benefits. For example, Telles et al. (2011) found that high-frequency yoga breathing significantly decreased the optical illusion, an indicator of improved attention and visual perception. Janakiramaiah et al. (1998) found that yoga breathing functioned as effectively as medical treatment for dysthymic disorder. Shastri et al. (2017) showed that yoga breathing reduced aggression, improved mindfulness, and emotion regulation in undergraduate students. Saoji et al. (2018) also found that yoga breathing enhanced psychological functions such as state mindfulness. Further, Tellhed et al. (2019) found mindfulness mediated the relationship between yoga breathing and mental health. Kizhakkeveettil et al. (2019) summarized that yoga breathing improved the quality of life in individuals with chronic disease by reducing stress, pain, anxiety, depression and fatigue, and improving sleep and emotion. Breathing, as an independent practice, was also found to impact on both autonomic and central nervous systems and psychological status such as increased comfort, vigor and alertness, and reduced symptoms of anxiety, anger, and confusion (Zaccaro et al., 2018).

As summarized above, both meditation and breathing have significant effects on biological and mental outcomes. Despite the usefulness of meditation and breathing, prior research suggests that they may function differently in influencing mental health outcomes such as energy (Joshi and Telles, 2009; Zaccaro et al., 2018) and attention (Brown and Gerbarg, 2009). First, breathing may be related to higher levels of energy, improved attention (Telles et al., 2011), increased available neural resources (Joshi and Telles, 2009), vigor and alertness (Zaccaro et al., 2018), while meditation is usually related to serenity (calmness of mind and body) and relaxation (Koopmann-Holm et al., 2013; Jones et al., 2018; Philips et al., 2019). The increased mental resources and alertness by breathing may stimulate active engagement and improve readiness for tasks, which coincides with work intention. Work intention refers to a guide to purposeful action as it is a mental representation of the behavior an employee chooses to manifest (Ajen and Fishbein, 1980). Intention to continue one’s work reflects one’s energy level at work, so breathing-focused yoga practice may retain higher levels of work intention than meditation-focused yoga practice. Second, while both meditation and breathing could help achieve mindfulness (Saoji et al., 2018; Wielgosz et al., 2019), breathing may be more practical in achieving mindfulness, a state of consciousness emphasizing attention and awareness in the present (Brown and Ryan, 2003). For example, “many people who try to learn meditation cannot focus their minds,” “some find the practices difficult and austere…lack the patience to persist,” and “trying to meditate while under severe stress sometimes magnifies the subjective sense of distress” (Brown and Gerbarg, 2009, p. 56). However, “one can affect the mind and consciousness through manipulation of the breath” (Brown and Gerbarg, 2009, p. 55). Thus, breathing-focused yoga practice may be more effective to improve mindfulness (i.e., attention and awareness) in a group of yoga novices. Since higher energy levels and mindfulness can help people decrease stress (Tong et al., 2020), breathing-focused yoga practice may be also better to reduce stress than meditation in a group of yoga novices.

Furthermore, a few researches have simultaneously compared the functions of meditation and breathing as yoga components (Ross et al., 2012). Only one study to our knowledge has compared between these yoga practices (Wheeler et al., 2019), which found that yoga manipulation (i.e., poses, breathing, meditation, or listening to a lecture about yoga) was equally effective in reducing anxiety and improving mood but did not affect responses to stressors in a group of yoga novices. This study utilized only a 20-min intervention, which is not a full yoga session and may be not enough to show any different effects of meditation and breathing. A longer intervention with multiple sessions over several weeks is needed to indicate any potential difference and show whether this difference can be retained.

Therefore, the current research aims to explore the potential differences between yoga practices of meditation and breathing in energy and stress-related outcomes in undergraduates, novices with daily mental activities. We hypothesize that breathing-focused yoga practice would be more effective than meditation-focused yoga to promote work intention (1a), and mindfulness (1b), and to reduce stress perception (1c). The varying effects of meditation and breathing may be evident in novice yoga exercisers such as undergraduate students; however, this assumption needs to be tested through empirical evidence. Most undergraduate students have little to no experience of practicing yoga exercise, and they are considered a vulnerable population for mental health (Balon et al., 2015). Engaging in considerable mental work daily, undergraduates would need to accumulate mental resources and alertness at work and to focus. Examining undergraduate students’ work intention, mindfulness, and perceived stress, as a function of yoga practices (breathing- or meditation-focused), is important to their academic performance and mental health. We chose to study undergraduate students in this study also because prior yoga research has shown success in recruiting and retaining a relatively large sample of undergraduate students as participants (Shastri et al., 2017; Wheeler et al., 2019); thus we felt confident to recruit a sample of undergraduate students in this study.

## Materials and Methods

### Experimental Design and Procedure

A 12-weeks yoga intervention was conducted among undergraduate students with no prior experience with yoga. Participants were assigned into two groups of meditation- and breathing-focused yoga practice, randomly by class. During the semester, students attended a morning yoga class per week for 12 consecutive weeks following the prescribed intervention. Participants were asked to dress in comfortable clothing and avoid a meal after getting up before attending the class. Baseline surveys were conducted before intervention, and post-training surveys were conducted after the 12th session. Surveys were requested to be finished before leaving the class.

Baseline and posttraining data on work intention, mindfulness, and perceived stress were collected. Age, gender, and other medication, health, and previous exercise information were collected at baseline. Surveys were delivered online by Wechat link or QR-code. It is a research design with a between-subject variable (meditation-focused group, breathing-focused group) and a within-subject variable (pre, post).

### Participants

Undergraduate students (*N* = 120) from four yoga classes at an academically prestigious Chinese university completed a survey measuring their current work intention, mindfulness, and stress, both at the beginning and the end of one semester. There were 27–32 students in each class. Participants were invited by survey links and made decisions to participate in this study by their own. Students received course credit for their participation. Before data collection, the university’s institutional review board approved the research protocol for human subjects.

Power analysis showed that the repeated ANOVA with within-between interaction needed a sample size of 90 at the power level of 0.80 (α = 0.05 with effect size of 0.15 and correlation among repeated measures of 0.50). Survey invitations were delivered to 120 participants. Individuals who have regular practice of yoga or similar techniques in the previous year were excluded (Tong et al., 2020), and there were 101 valid data for pre-survey. The final sample, with matched pre- and post-intervention measures, included 86 students (with five males) aged between 19 and 23 (*M* = 20.79; *SD* = 1.00). The attrition rate is 71.7%, and there is no significant difference on age or gender between the attrition and remained group. A total of 46 participants from two yoga classes were designated into the breathing-focused yoga group, while 40 participants from the other two yoga classes were designated into the meditation-focused yoga group. These two groups did not differ on demographic (i.e., age, gender) or pre-intervention measures (i.e., work intention, mindfulness, stress), based on *t*-tests analyses.

### Hatha Yoga Intervention Program

Usually, one-time breathing intervention lasts for 1–20 min (Joshi and Telles, 2009; Telles et al., 2011; Saoji et al., 2018; Tellhed et al., 2019), and yoga exercise may be more effective when different parts function together. Thus, our study aimed to utilize a normal 80-min yoga session with a valid 10-min practice of meditation/breathing and compare the relative importance of meditation and breathing across 12 sessions of yoga practices. To conduct a fair comparison, the participants would be requested to breathe at a low rate similar to mediation, but breathing deeply into the abdomen, and pursue and develop awareness of in-and-out breathing (Brown and Gerbarg, 2009). Although meditation can be divided into two categories as focusing on mental processes or focusing on bodily processes (Hendriks, 2018), meditation-focused yoga practice in this study focused on the mental processes including focused attention, open monitoring, and visualization (Jindal et al., 2013).

Participants performed 80 min of Hatha yoga exercise (Riley and Park, 2015) in a morning yoga class per week for 12 weeks. Each class comprised of three main stages, including meditation or breathing (10 min, Stage 1), posture-holding exercise (60 min, Stage 2), and relaxation (10 min, Stage 3), as shown in Table 1. Students were directed to do meditations in the meditation-focused yoga group (“Please adjust your posture and close your eyes…Imagine some pictures you like…Gaze at the picture in details and keep focused…Draw back your attention when realizing you get distracted…Feel your inner calm state…”) while doing breathing in the breathing-focused yoga group (“Please adjust your posture and close your eyes…Keep breathing slowly and deeply…Observe your own breathing deep into your chest, into your abdomen…Feel the movement of diaphragm and abdomen wall and the extension of the spine…Visualizing the breath moving up and down and in and out your body…Feel the temperature of our breath in and out…Feel the steady rhythm of smooth breathing…”) during Stage 1 for each class. Participants in the meditation-focused yoga group were instructed to engage in imagination of nice and vivid pictures in mind with voluntarily focused attention and non-reactive open monitoring of the contents of own experience (Jindal et al., 2013). Participants in the breathing-focused yoga group were instructed to engage in a slow and rhythmic breathing performed deeply into the abdomen and through the nostrils (Brown and Gerbarg, 2009), indicated as initial breath regulation in Hatha yoga, focus their own attention on the breath (Kabat-Zinn, 2003), and pursue and develop awareness of in-and-out breathing (Brown and Gerbarg, 2009).

**TABLE 1:** Hatha Yoga intervention program.

Stage (time)	Breathing-focused group	Meditation-focused group
1. Intervention (10 min)	Breathing: Participants were instructed to engage in slow and rhythmic breathing performed deeply into the abdomen and through the nostrils (Brown and Gerbarg, 2009), as indicated as initial breath regulation in Hatha yoga, focus attention on the breathing (Kabat-Zinn, 2003) and pursue and develop awareness of in-and-out breathing (Brown and Gerbarg, 2009).…“Please adjust your posture and close your eyes…Continue to breathe slowly and deeply…Observe breathing deep into your chest, into your abdomen…Feel the movement of diaphragm and abdomen wall and the extension of the spine…Visualize the breath moving up and down and in and out your body…Feel the temperature of our breath in and out…Feel the steady rhythm of smooth breathing…”	Mediation: Participants were instructed to engage in imagination of nice and vivid pictures in mind with voluntarily focused attention and non-reactive open monitoring of the contents of own experience (Jindal et al., 2013). “Please adjust your posture and close your eyes…Imagine some pictures you like…Gaze at the picture in details and keep focused…Draw back your attention when realizing you are distracted…Feel your inner calm state…”
2. Posture (60 min)	Posture-holding includes 10–12 postures after warm-up such as forward folding, bridge pose, cobra, bow, waist rotating, downward facing dog, cat stretch, warrior, triangle, tree. Practice 6–10 times for each posture. Stay in each posture for 30–60 s.	
3. Relaxation (10 min)	Relaxation means to lie in tranquil, stop any physical and mental activities, and relax each part of the body. The instructor speaks out names of specific body parts to lead the scanning relaxation. Finally, the instructor describes one or two pictures with relaxation and calm.	

The intervention plan was strictly followed in each session. The participants had full participation and involvement in each session of the yoga practices. The attendance rate for all sessions was 100%.

### The Instructor

These interventions were provided by the same instructor using the same instructions and the same music across the two conditions each week. The instructor was the first author of this paper, who designed the schema for yoga intervention. She was a female tenured professor in the department of physical education with more than 13 years of experiences teaching and coaching yoga to undergraduate students. She was a senior fitness yoga trainer certified by the Federation of University Sports of China. She also held certification in instructing Yogi Yoga, a registered yoga school by Yoga Alliance USA. Additionally, her massive open online yoga course (MOOC) achieved the national online open course certification from the National Department of Education, which is also listed in the global online course platform of Coursera and is selected by over 220,000 students.

### Instrumentations

#### Demographic Survey

Participants were requested to fill in the blanks with their student ID and age. They were asked to choose the class they took and their gender.

#### Work Intention

Work intention was measured by six items in both pre- and post-training surveys. It was developed for this research according to the definition of work intention (Ajen and Fishbein, 1980). Participants were requested to rate on a seven-point scale ranging from 1 (very much unwilling) to 7 (very much willing) about their willingness to complete the survey. A sample item is phrased as “please indicate how much you want to continue completing the remaining survey right now.” The reliabilities are αs = 0.97 and 0.98 for pre- and post-interventions, respectively.

#### Mindfulness

Mindfulness was measured by Brown and Ryan’s (2003) 15-item Mindful Attention and Awareness Scale on a seven-point scale ranging from 1 (never) to 7 (always). Participants were requested to rate how frequently each situation occurs recently, rather than whether they generally agree. A sample item is stated as “It seems I am ‘running on automatic’ without much awareness of what I’m doing” (reverse scored). The reliabilities are αs = 0.86 and 0.91 for pre- and post-interventions, respectively.

#### Perceived Stress

Stress was measured by seven items adopted from the Depression Anxiety Stress Scale (Lovibond and Lovibond, 1995) on a seven-point scale ranging from 1 (never) to 7 (always). The instruction for administering this survey was the same with the mindfulness measure. It was used in previous yoga literature (Tong et al., 2020). Sample items include, “I found it difficult to relax” and “I tended to over-react to situations.” The reliabilities are αs = 0.89 and 0.90 for pre- and post-interventions, respectively.

### Data Analysis

We conducted a repeated-measure multivariate analysis of covariance (MANCOVA), followed by univariate analyses, to examine the differences between meditation-focused and breathing-focused yoga interventions over the semester, in terms of ratings of work intention, mindfulness, and stress. Many previous yoga researches focused on a designated group of age and sex (such as middle-aged women, adolescence, undergraduates, see Quach et al., 2016; Philips et al., 2019). Some research explored how age or sex were related to yoga practice effect (Savita, 2006; Cahn et al., 2017; Rojiani et al., 2017) and some controlled demographic variables in empirical studies (Fishbein et al., 2016; Cahn et al., 2017; Tong et al., 2020). Moreover, age and/or sex were found to be significantly related to outcomes of work motivation (Boumans et al., 2012), mindfulness (Edwards, 2019), and stress (Folkman et al., 1987). Thus, age and gender were included as covariates^1^ in this model. Pre- and post-measures were analyzed as within-group variables, and the intervention type was the between-group variable.

## Results

Table 2 presents the descriptive statistical results for all research variables over time for each intervention group, and Table 3 presents the correlation matrix. The intra-class correlations (ICCs) for all focal variables were small (ranging 0.01–0.03), indicating that there was none to minimal clustering effect and that the data observations for these focal constructs were independent despite class memberships.

**TABLE 2:** Descriptive statistics for each group, mean (SD).

Variable	Breathing-focused group	Meditation-focused group
Age	20.63 (1.10)	20.98 (0.83)
Gender (% women)	95.65%	92.50%
*N*	46	40
**Pre-intervention**		
Work intention	4.48 (1.49)	4.36 (1.49)
Mindfulness	4.50 (0.89)	4.50 (0.91)
Stress	3.87 (1.26)	3.73 (1.33)
**Post-intervention**		
Work intention	4.36 (1.59)	3.56 (1.64)
Mindfulness	4.90 (1.04)	4.45 (1.04)
Stress	3.29 (1.21)	3.74 (1.26)

**TABLE 3:** Correlations between research variables.

Variable	1	2	3	4	5	6	7	8	9
1 Intervention group	–								
2 Pre-work intention	–0.04	–							
3 Pre-mindfulness	0.001	0.15	–						
4 Pre-stress perception	–0.06	0.03	−0.56**	–					
5 Post-work intention	−0.24*	0.56**	0.15	0.02	–				
6 Post-mindfulness	−0.21*	0.07	0.59**	−0.32**	0.32**	–			
7 Post-stress perception	0.18	0.05	−0.42**	0.36**	−0.24*	−0.72**	–		
8 Age	0.17	0.02	–0.15	0.03	–0.01	–0.07	–0.04	–	
9 Sex	0.07	−0.29**	–0.03	0.01	–0.17	–0.06	0.06	−0.30**	–

N = 86. *p < 0.05, **p < 0.01.

**FIGURE 1:**
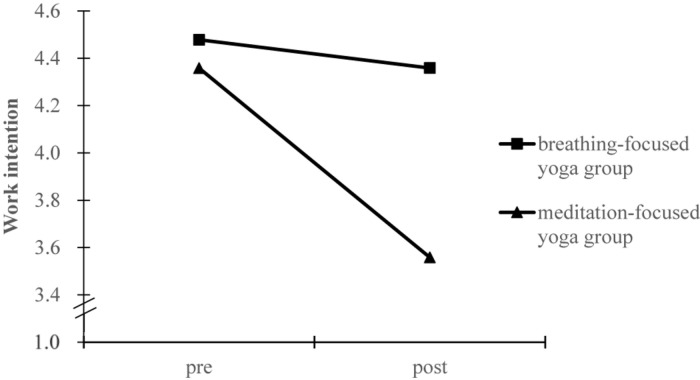
Marginal means estimated for work intention before and after intervention by group.

**FIGURE 2:**
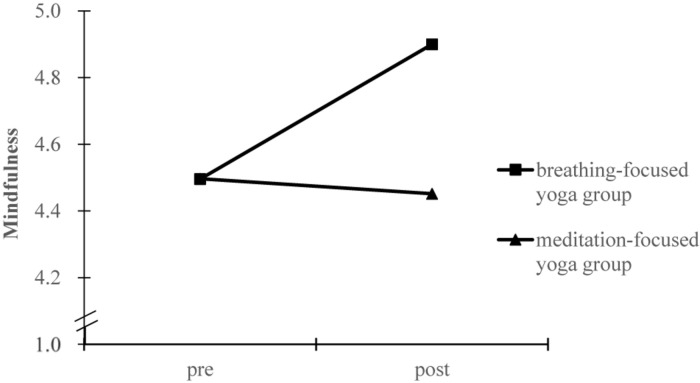
Marginal means estimated for mindfulness before and after intervention by group.

**FIGURE 3:**
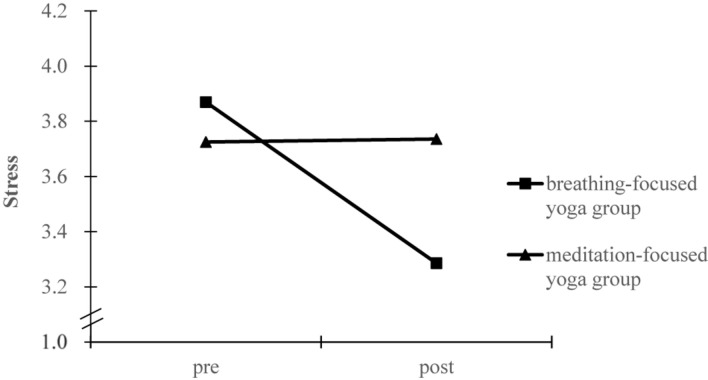
Marginal means estimated for stress before and after intervention by group.

The test of homogeneity of covariance matrices was not significant [Box’s *M* = 25.81, *F*_(21, 24883)_ = 1.13, *p* = 0.30], indicating that the observed covariance matrices of the dependent variables were equal across groups. Results from the pre-post training MANCOVA revealed significant group differences with a median effect size [Wilks’ lambda, Λ = 0.90, *F*(3, 80) = 3.10, *p* = 0.031, η^2^ = 0.104]. Subsequently, significant group by time interaction effects were observed for work intention [*F*_(1, 82)_ = 5.22, *p* = 0.025, η^2^_*p*_ = 0.060), mindfulness [*F*_(1, 82)_ = 6.33, *p* = 0.014, η^2^_*p*_ = 0.072], and stress [*F*_(1, 82)_ = 4.20, *p* = 0.044, η^2^_*p*_ = 0.049], respectively. To characterize the meaning of these interactions, marginal means of the models were estimated for each group at pre and post measures, as shown in Figures 1–3. Simple effect analyses showed that between-group differences emerged at post-intervention for work intention [*t*(84) = 2.30, *p* = 0.024], mindfulness [*t*(84) = 2.00, *p* = 0.049], and stress [*t*(84) = −1.68, *p* = 0.096]. Students in the breathing-focused group had significant higher work intentions and mindfulness but marginally lower stress than students in the meditation-focused group. Examination of the change for each intervention group also supported the function of breathing-focused yoga intervention. Comparatively, the breathing-focused group had significant increase in mindfulness (B_breathing_ = 0.42 ± 0.13, *p* = 0.002; B_meditation_ = −0.07 ± 0.14, *p* = 0.64) and decrease in stress (B_breathing_ = −0.61 ± 0.21, *p* = 0.005; B_meditation_ = 0.04 ± 0.23, *p* = 0.87) over the semester. The meditation-focused group significantly decreased work intention (B_breathing_ = −0.09 ± 0.22, *p* = 0.66; B_meditation_ = −0.83 ± 0.23, *p* = 0.001).

## Discussion

The aim of this research was to compare the relative importance of meditation-focused and breathing-focused yoga practice in energy retention and related stress reduction in a group of undergraduate students. Major findings are: (1) Students in the breathing-focused yoga group reported significant increase in mindfulness and significant decrease in stress, while students in the meditation-focused yoga group reported significant decrease in work intention; (2) Students in the breathing-focused yoga group had significant higher work intentions and mindfulness and had marginally less stress than students in the meditation-focused yoga group at post-intervention.

It was found that students in the meditation-focused yoga group reported significant decrease in work intention, while the breathing-focused group did not have any significant changes in terms of work intention. It may be due to the time for the post-training survey was in the end of a normal semester, and students were busy coping with the final exams. With much resources invested into the final exams, students may have limited energy to spend in other tasks such as filling out a survey. It can be indicated as lower levels of purposeful action (Ajen and Fishbein, 1980).

We found that breathing-focused yoga practice is more effective than meditation-focused yoga practice in increasing mindfulness and reducing stress. It is different from previous no-difference findings (Wheeler et al., 2019). As discussed in the Introduction section, a 20-min intervention without a full yoga intervention (combined with postures) and without an intervention of multiple sessions may limit the function of breathing and meditation (Wheeler et al., 2019). Our findings are consistent with previous literature supporting that breathing directly increase oxygenation to strengthen the physical body (Brown and Gerbarg, 2005) and achieves energy and resources (Joshi and Telles, 2009; Telles et al., 2011; Zaccaro et al., 2018), while meditation achieves serenity and relaxation (Koopmann-Holm et al., 2013; Jones et al., 2018; Philips et al., 2019). When people more easily achieve energy and resources through breathing-focused yoga practice, they would not frequently feel stressed, compared to meditation-focused practice. Moreover, breathing may be more practical and is related to vigor and alertness (Zaccaro et al., 2018), while meditation may be not easily learned and practiced for undergraduates (Brown and Gerbarg, 2009). Thus, breathing may easily help undergraduates achieve better attention and awareness (i.e., mindfulness), which also leads to lower stress. Although our findings may be due to the relatively easily achieved benefits of yoga breathing and biological energy enhancement by better oxygenation, it may be also due to the difference between awareness of inner body and outer imagination. When people are inexperienced yoga practitioners, practice of mental attention on inner body may be easier to heighten the level of spiritual awareness than through outer imagination. However, all the mentioned benefits of mediation (Hendriks, 2018) may be achieved when people are experienced.

The present research contributes to the yoga literature by going further in disclosure the nature of important yoga components. It provided evidence showing the advantage of breathing-focused yoga over meditation-focused yoga practice in terms of energy retention, attention and awareness, and stress reduction, which confirms the value to direct a nuanced examination of separate yoga components. This stream of research would indicate the relative importance of yoga components and disclose the nature of these components. Practically, our findings would help novices find a quick and easy way to achieve benefits of yoga, as breathing is easier than meditation (Brown and Gerbarg, 2009). Practitioners can take into separate yoga components into detailed consideration when designing a new yoga program. For example, postures with more breathing and less meditation may be an effective yoga program for young and inexperienced people.

Besides the above strengths, there are some limitations. First, all the indicators were self-reported. The benefits of yoga components may be subjective rather than objective. Future research may use other ratings or biological indicators such as cortisol and event-related potentials (P300) to better support the function of individual yoga components. Since breathing regulation involves both biological techniques and mental awareness (Ramdev, 2005) and meditation involves both mental and bodily processes (Hendriks, 2018), the biological indicators may also help to examine detailed components of breathing and meditation. Second, meditation may be better conducted in older adults such as the working population with richer personal experience than undergraduate students. Future research needs to explore whether our findings can be generalized into other groups.

Another limitation of the study is with the measurement of work intention. Work intention is an indication of energy, which was hypothesized to be an important psychological benefit of yoga practice, especially of breathing-focused yoga practice. The measure showed sound internal consistency reliability in this current study. However, asking the participants to reference to an immediate cognitive task (i.e., completing the survey) as representation of work may not thoroughly capture undergraduate students’ work or occupation in general, although completing the survey was the immediate task they were asked to perform. Future research may examine whether yoga would energize undergraduate students’ work intention in other tasks (e.g., studying in a course, working for a part-time job, cramming for an exam).

Finally, lack of quality of delivery data should be recognized as an area of weakness for fidelity check in this study. Future research is encouraged to collect data about participants’ effort and attentions.

## Conclusion

Yoga breathing is an important component and can achieve better benefits than yoga mediation in undergraduate students. Designing a yoga program with combination of breathing and postures facilitates psychological resources and stress coping.

## Data Availability Statement

The original contributions presented in the study are included in the article/Supplementary Material, further inquiries can be directed to the corresponding author.

## Ethics Statement

The studies involving human participants were reviewed and approved by the Committee for Protecting Human and Animal Subjects, School of Psychological and Cognitive Sciences, Peking University. The ethics committee waived the requirement of written informed consent for participation.

## Author Contributions

XQ, JT, ZH, and SC contributed to conception and design of the study. XQ conducted the experiment. JT, XZ, and SC analyzed the data. JT wrote the first draft of the manuscript. All authors contributed to manuscript revision and read and approved the submitted version.

## Conflict of Interest

The authors declare that the research was conducted in the absence of any commercial or financial relationships that could be construed as a potential conflict of interest.

## Footnotes

1 Since there were no age and gender differences between groups in this research, we also analyzed the data without any controls. Without controlling for age and gender, the MANOVA and ANOVAs results are very similar. Results from the pre-post training MANOVA revealed significant group differences with a median effect size (Wilks’ lambda, Λ = 0.91, F(3.82) = 2.81, p = 0.045,η^2^ = 0.093). Subsequently, group by time interaction effects were observed significant for work intention (F_1,84_ = 4.74,p = 0.032, η^2^_*p*_ = 0.053), mindfulness (F _1,84_ = 5.72, p = 0.019, η^2^_*p*_ = 0.064), and marginally for stress (F_1,84_ = 3.81, p = 0.054, η^2^_*p*_ = 0.043), respectively.

## References

[B1] Ajen I. Fishbein M. (1980). *Understanding Attitudes and Predicting Social Behavior.* Englewood Cliffs, NJ: Prentice-Hall.

[B2] Balon R. Beresin E. V. Coverdale J. H. Louie A. K. Roberts L. W. (2015). College mental health: a vulnerable population in an environment with systemic deficiencies. *Acad. Psychiatry.* 39 495–497. 10.1007/s40596-015-0390-1 26327172

[B3] Borchardt A. R. Zoccola P. M. (2018). Recovery from stress: an experimental examination of focused attention meditation in novices. *J. Behav. Med.* 41 836–849. 10.1007/s10865-018-9932-9 29850971

[B4] Boumans N. P. G. de Jong A. H. J. Janssen S. M. (2012). Age-differences in work motivation and job satisfaction: the influence of age on the relationship between work characteristics and workers’ outcomes. *Int. J. Aging Hum. Dev.* 73 331–350. 10.2190/ag.73.4.d 22474915

[B5] Bowden D. Gaudry C. An S. C. Gruzelier J. (2012). A comparative randomised controlled trial of the effects of brain wave vibration training, iyengar yoga, and mindfulness on mood, well-being, and salivary cortisol. *Evid. Based Complement Alternat. Med.* 2012:234713. 10.1155/2012/234713 22216054 PMC3246835

[B6] Brandani J. Z. Mizuno J. Ciolac E. G. Monteiro H. L. (2017). The hypotensive effect of Yoga’s breathing exercises: a systematic review. *Complement Ther. Clin. Pract.* 28 38–46. 10.1016/j.ctcp.2017.05.002 28779935

[B7] Brown K. W. Ryan R. M. (2003). The benefits of being present: mindfulness and its role in psychological well-being. *J. Pers. Soc. Psychol.* 84 822–848. 10.1037/0022-3514.84.4.822 12703651

[B8] Brown R. P. Gerbarg P. L. (2005). Sudarshan Kriya Yogic breathing in the treatment of stress, anxiety, and depression. Part II–clinical applications and guidelines. *J. Altern. Complement Med.* 11 711–717. 10.1089/acm.2005.11.711 16131297

[B9] Brown R. P. Gerbarg P. L. (2009). Yoga breathing, meditation, and longevity. *Ann. N. Y. Acad. Sci.* 1172 54–62. 10.1111/j.1749-6632.2009.04394.x 19735239

[B10] Cahn B. R. Goodman M. S. Peterson C. T. Maturi R. Mills P. J. (2017). Yoga, meditation and mind-body health: increased BDNF, cortisol awakening response, and altered inflammatory marker expression after a 3-month yoga and meditation retreat. *Front. Hum. Neurosci.* 11:315. 10.3389/fnhum.2017.00315 28694775 PMC5483482

[B11] Carter J. Byrne G. (2004). *A Two Year Study of the use of Yoga in a Series of Pilot Studies as an Adjunct to Ordinary Psychiatric Treatment in a Group of Vietnam War Veterans Suffering From Post Traumatic Stress Disorder.* Available at: https://www.Therapywithyoga.com (Accessed November 27, 2004).

[B12] Chiesa A. Calati R. Serretti A. (2011). Does mindfulness training improve cognitive abilities? A systematic review of neuropsychological findings. *Clin. Psychol. Rev.* 31 449–464. 10.1016/j.cpr.2010.11.003 21183265

[B13] Clarke T. C. Barnes P. M. Black L. I. Stussman B. J. Nahin R. L. (2018). Use of yoga, meditation, and chiropractors among U.S. adults aged 18 and over. *NCHS Data Brief.* 325 1–8. 30475686

[B14] Edwards D. J. (2019). Age, pain intensity, values-discrepancy, and mindfulness as predictors for mental health and cognitive fusion: hierarchical regressions with mediation analysis. *Front. Psychol.* 10:517. 10.3389/fpsyg.2019.00517 30899236 PMC6416201

[B15] Fishbein D. Miller S. Herman-Stahl M. Williams J. Lavery B. Markovitz L. (2016). Behavioral and psychophysiological effects of a yoga intervention on high-risk adolescents: a randomized control trial. *J. Child. Fam. Stud.* 25 518–529. 10.1007/s10826-015-0231-6

[B16] Folkman S. Lazarus R. S. Pimley S. Novacek J. (1987). Age differences in stress and coping processes. *Psychol. Aging* 2 171–184. 10.1037/0882-7974.2.2.171 3268206

[B17] Gallegos A. M. Crean H. F. Pigeon W. R. Heffner K. L. (2017). Meditation and yoga for posttraumatic stress disorder: a meta-analytic review of randomized controlled trials. *Clin. Psychol. Rev.* 58 115–124. 10.1016/j.cpr.2017.10.004 29100863 PMC5939561

[B18] Hendriks T. (2018). The effects of Sahaja Yoga meditation on mental health: a systematic review. *J. Complement Integr. Med.* 15:20160163. 10.1515/jcim-2016-0163 29847314

[B19] Hilton L. Maher A. R. Colaiaco B. Apaydin E. Sorbero M. E. Booth M. (2017). Meditation for posttraumatic stress: systematic review and meta-analysis. *Psychol. Trauma* 9 453–460. 10.1037/tra0000180 27537781

[B20] Janakiramaiah N. Gangadhar B. N. Murthy P. Harish M. G. Shetty K. T. Subbakrishna D. K. (1998). Therapeutic efficacy of sudarshan kriya yoga (sky) in dysthymic disorder. *Nimhans J.* 16 21–28.

[B21] Jindal V. Gupta S. Das R. (2013). Molecular mechanisms of meditation. *Mol. Neurobiol.* 48 808–811. 10.1007/s12035-013-8468-9 23737355

[B22] Jones D. R. Graham-Engeland J. E. Smyth J. M. Lehman B. J. (2018). Clarifying the associations between mindfulness meditation and emotion: daily high- and low-arousal emotions and emotional variability. *Appl. Psychol. Health Well Being* 10 504–523. 10.1111/aphw.12135 29992747

[B23] Joshi M. Telles S. (2009). A nonrandomized non-naive comparative study of the effects of kapalabhati and breath awareness on event-related potentials in trained yoga practitioners. *J. Altern. Complement Med.* 15 281–285. 10.1089/acm.2008.0250 19243275

[B24] Kabat-Zinn J. (2003). Mindfulness-based interventions in Context: past, present, and future. *Clin. Psychol. Sci. Pract.* 10 144–156. 10.1093/clipsy.bpg016

[B25] Kizhakkeveettil A. Whedon J. Schmalzl L. Hurwitz E. L. (2019). Yoga for quality of life in individuals with chronic disease: a systematic review. *Altern. Ther. Health Med.* 25 36–43. 30982785

[B26] Koopmann-Holm B. Sze J. Ochs C. Tsai J. L. (2013). Buddhist-inspired meditation increases the value of calm. *Emotion* 13 497–505. 10.1037/a0031070 23356567

[B27] Lovibond P. F. Lovibond S. H. (1995). The structure of negative emotional states: comparison of the depression anxiety stress scales (DASS) with the beck depression and anxiety inventories. *Behav. Res. Ther.* 33 335–343. 10.1016/0005-7967(94)00075-u 7726811

[B28] Philips K. H. Brintz C. E. Moss K. Gaylord S. A. (2019). Didgeridoo sound meditation for stress reduction and mood enhancement in undergraduates: a randomized controlled trial. *Glob. Adv. Health Med.* 8 1–10. 10.1177/2164956119879367 31632840 PMC6769210

[B29] Quach D. Jastrowski Mano K. E. Alexander K. (2016). A randomized controlled trial examining the effect of mindfulness meditation on working memory capacity in adolescents. *J. Adolesc. Health* 58 489–496. 10.1016/j.jadohealth.2015.09.024 26576819

[B30] Ramdev S. (2005). *Pranayama: Its Philosophy and Practice.* Haridwar: Divya Prakashan.

[B31] Riley K. E. Park C. L. (2015). How does yoga reduce stress? A systematic review of mechanisms of change and guide to future inquiry. *Health Psychol. Rev.* 9 379–396. 10.1080/17437199.2014.981778 25559560

[B32] Rojiani R. Santoyo J. F. Rahrig H. Roth H. D. Britton W. B. (2017). Women benefit more than men in response to college-based meditation training. *Front. Psychol.* 8:551. 10.3389/fpsyg.2017.00551 28473783 PMC5397480

[B33] Ross A. Friedmann E. Bevans M. Thomas S. (2012). Frequency of yoga practice predicts health: results of a national survey of yoga practitioners. *Evid. Based Complement Altern. Med.* 2012:983258. 10.1155/2012/983258 22927885 PMC3425136

[B34] Saoji A. A. Raghavendra B. R. Madle K. Manjunath N. K. (2018). Additional practice of yoga breathing with intermittent breath holding enhances psychological functions in yoga practitioners: a randomized controlled trial. *Explore* 14 379–384. 10.1016/j.explore.2018.02.005 30122326

[B35] Savita R. K. (2006). *Comparehensive Training of Astang Yoga on Reaction Time and Selected Physiological Variables in Relation to Age and Sex of School Children.* Doctroal thesis, Lakshmibai National Institute of Physical Education, Gwalior.

[B36] Schmalzl L. Powers C. Zanesco A. P. Yetz N. Groessl E. J. Saron C. D. (2018). The effect of movement-focused and breath-focused yoga practice on stress parameters and sustained attention: a randomized controlled pilot study. *Conscious Cogn.* 65 109–125. 10.1016/j.concog.2018.07.012 30099318

[B37] Shastri V. V. Hankey A. Sharma B. Patra S. (2017). Investigation of yoga pranayama and vedic mathematics on mindfulness, aggression and emotion regulation. *Int. J. Yoga* 10 138–144. 10.4103/0973-6131.213470 29422744 PMC5793008

[B38] Shonin E. Van Gordon W. Griffiths M. D. (2014). Meditation awareness training (MAT) for improved psychological well-being: a qualitative examination of participant experiences. *J. Relig. Health* 53 849–863. 10.1007/s10943-013-9679-0 23377964

[B39] Telles S. Maharana K. Balrana B. Balkrishna A. (2011). Effects of high-frequency yoga breathing called kapalabhati compared with breath awareness on the degree of optical illusion perceived. *Percept. Mot. Skills* 112 981–990. 10.2466/02.20.22.PMS.112.3.981-990 21853784

[B40] Tellhed U. Daukantaitë D. Maddux R. E. Svensson T. Melander O. (2019). Yogic breathing and mindfulness as stress coping mediate positive health outcomes of yoga. *Mindfulness* 10 2703–2715. 10.1007/s12671-019-01225-4

[B41] Tong J. Qi X. He Z. Chen S. Pederson S. J. Cooley P. D. (2020). The immediate and durable effects of yoga and physical fitness exercises on stress. *J. Am. Coll. Heath* 1–9. 10.1080/07448481.2019.1705840 31944898

[B42] Tyagi A. Cohen M. Reece J. Telles S. Jones L. (2016). Heart rate variability, flow, mood and mental stress during yoga practices in yoga practitioners, non-yoga practitioners and people with metabolic syndrome. *Appl. Psychophysiol. Biofeedb.* 41 381–393. 10.1007/s10484-016-9340-2 27457341

[B43] Wheeler E. A. Santoro A. N. Bembenek A. F. (2019). Separating the “limbs” of yoga: limited effects on stress and mood. *J. Relig. Health* 58 2277–2287. 10.1007/s10943-017-0482-1 28819762

[B44] Wielgosz J. Goldberg S. B. Kral T. R. A. Dunne J. D. Davidson R. J. (2019). Mindfulness meditation and psychopathology. *Annu. Rev. Clin. Psychol.* 15 285–316. 10.1146/annurev-clinpsy-021815-093423 30525995 PMC6597263

[B45] Zaccaro A. Piarulli A. Laurino M. Garbella E. Menicucci D. Neri B. (2018). How breath-control can change your life: a systematic review on psycho-physiological correlates of slow breathing. *Front. Hum. Neurosci.* 12:353. 10.3389/fnhum.2018.00353 30245619 PMC6137615

